# A systematic review on diagnostics and surgical treatment of adult right-sided Bochdalek hernias and presentation of the current management pathway

**DOI:** 10.1007/s10029-021-02445-1

**Published:** 2021-07-03

**Authors:** J. P. Ramspott, T. Jäger, M. Lechner, P. Schredl, A. Gabersek, F. Mayer, K. Emmanuel, S. Regenbogen

**Affiliations:** 1 Department of Surgery, Paracelsus Medical University Salzburg, Müllner Hauptstraße 48, 5020 Salzburg, Austria; 2 Department of Gynecology and Obstetrics, Münster University Hospital, Münster, Germany; 3 Department of Trauma Surgery, BG Trauma Center Murnau, Murnau, Germany

**Keywords:** Bochdalek hernia, Hernia, Algorithm, Repair, Mesh, Suture

## Abstract

**Purpose:**

Bochdalek hernia is a congenital diaphragmatic hernia. The incidence in adults is estimated around 0.17%. Right-sided hernias are much more seldom than left-sided ones because of faster closure of the right pleuroperitoneal canal and the protective effect of the liver. Due to its rarity, there have been no large prospective or retrospective studies following great need for evidence-based diagnostics and treatment strategies. In this systematic review, we evaluated the current evidence of diagnostics, treatment, and follow-up of adult right-sided Bochdalek hernias.

**Methods:**

According to the Preferred Reporting Items for Systematic Reviews and Meta-Analyses (PRISMA) guidelines a systematic literature review was conducted in PubMed and Cochrane library from 2004 to January 2021. The literature search included all studies with non-traumatic right-sided Bochdalek hernias. Literature on left- or both-sided, pregnancy-associated, pediatric, and other types of hernias were explicitly excluded. Quality assessment of the included studies was performed.

**Results:**

Database search identified 401 records. After eligibility screening 41 studies describing 44 cases of right-sided non-traumatic Bochdalek hernias in adulthood were included for final analysis. Based upon the systematic literature review, the current diagnostic, therapeutic, and follow-up management pathway for this rare surgical emergency is presented.

**Conclusion:**

This systematic review underlined that most studies investigating management of adult non-traumatic right-sided Bochdalek hernias are of moderate to low methodological quality. Hernias tend to occur more frequently in middle-aged and older women presenting with abdominal pain and dyspnea. A rapid and accurate diagnosis following surgical repair and regular follow-up is mandatory. High-quality studies focusing on the management of this rare entity are urgently needed.

**Supplementary Information:**

The online version contains supplementary material available at 10.1007/s10029-021-02445-1.

## Introduction

Diaphragmatic hernias may occur congenitally or secondarily due to trauma allowing thoracic herniation of abdominal contents. Normally, diaphragmatic formation separates the thoracic cavity from the peritoneal one around week 8 of gestation [1]. Diaphragmatic hernias have been classified into posterolateral, anterior, or central [2]. The posterolateral defect in the lumbocostal triangle (Bochdalek hernia) is the most common type, which was first described by Vincent Alexander Bochdalek in 1848. It is caused by a persistence of the pleuroperitoneal cavity and mostly an incidental finding in children. Congenital diaphragmatic hernias have an incidence of approximately one per 2500 births of whom 70 to 75% are classified as Bochdalek hernias [3]. Genetic and environmental factors are involved in the development of hernias and they mostly cause respiratory symptoms directly after birth [4, 5]. The incidence of adult Bochdalek hernias is estimated around 0.17% [6]. Right-sided Bochdalek hernias are less common than left-sided ones due to faster closure of the right pleuroperitoneal canal and the protective effect of the liver [7]. They mostly present with gastrointestinal or respiratory complaints or asymptomatically. Increased intraabdominal pressure and previous abdominal surgery may be precipitating [7, 8]. Mortality rates in children vary between 42 and 68% [9, 10], whereas there are only rare data in adults. In contrast to the affected children [11], a clear diagnostic, therapeutic, and follow-up pathway for right-sided Bochdalek hernias in adulthood is missing so far. Due to its rarity, there have been no large retrospective or prospective studies. As these patients mostly present in an emergency setting, there is great need for evidence-based diagnostics and treatment strategies as right-sided Bochdalek hernias are rarely investigated as a separate entity. To the best of our knowledge, no systematic literature review of this rare entity is available to date. In this systematic review, we evaluated the current evidence of published studies describing adult right-sided Bochdalek hernias and evaluated the main patients’ characteristics, clinical presentations, and different treatment approaches. Based upon our systematic literature review, we present the current diagnostic, therapeutic, and follow-up management pathway for this rare surgical emergency.

## Methods

### Search strategy

A systematic literature review was conducted in line with the Preferred Reporting Items for Systematic Reviews and Meta-Analyses (PRISMA) guidelines [12]. The electronic databases searched included PubMed and Cochrane library. The search strategy included the following key words: ‘right-sided Bochdalek hernia’ OR ‘right-sided diaphragmatic hernia’ from 2004 to 2020. Rout et al. described all cases of adult right-sided Bochdalek hernias published until 2004 [13]. The final search was conducted on 31st of January 2021. Additionally, a snowball search of the included references was performed [14].

### Study selection

In this systematic review only research papers were included if the following inclusion criteria were fulfilled: right-sided non-traumatic Bochdalek hernias and patient’s age ≥ 18 years. Articles describing left- or both-sided Bochdalek hernias, other types of hernias, pregnancy-associated Bochdalek hernias, fetal studies, and traumatic Bochdalek hernias were excluded. Articles not written in English, conference papers, and animal studies were also excluded from this systematic review. First, titles and abstracts were scanned according to the above defined eligibility criteria. Finally, full-text evaluation was performed. In total, 41 studies with 44 cases of right-sided non-traumatic Bochdalek hernias in adults could be identified and included into the analysis (Table 1). No cohort studies or randomized controlled trials were identified.

**Table 1:** Summary of reported non-traumatic right-sided Bochdalek hernias in adults published 2004–2020

No	References	Age/sex	Chief complaint	Past medical history	Diagnostic imaging	Surgical approach	Hernia orifice (cm)	Closure	Herniated organ	Bowel resection	Complication	Outcome
1	Gupta et al. [39]	78/M	Right-sided chest pain, dyspnea	Unremarkable	CT	Laparotomy	2 × 2	Suture (NA)	Small bowel	Small bowel	Lung empyema (HR)	N/A
2	Lau et al. [32]	77/M	Abdominal pain, right flank pain	N/A	CT	Laparoscopy	8	Suture (NA), mesh (NA)	Kidney, perinephric fat	No	Hydronephrosis (HR)	No recurrence at 2 months
3	Lau et al. [32]	42/F	Dyspnea, shoulder pain	N/A	CT	Laparoscopy, thoracoscopy	12 × 5	Suture (NA), mesh (NA)	Liver, kidney, colon	No	None	No recurrence at 2 months
4	Lau et al. [32]	66/M	Abdominal pain	Renal transplant	CT	Laparoscopy, laparotomy	10 × 5	Suture (N/A), mesh (A)	Small bowel, colon, omentum	Colon	Colonic ischemia (HR)	No recurrence at 12 months
5	Nassiri et al. [23]	82/F	Right-sided chest pain, right shoulder pain, flank pain	Hypertension, diabetes	CT	Right-sided double-J ureteral stent, recommendation of outpatient surgical repair	5.4 × 8.2 × 6.9	None	Fat, extra-renal pelvis, ureter	No	Hydronephrosis (HR)	N/A
6	Rocha Paiva et al. [37]	92/M	Thoracic pain, dyspnea, fever	COPD, hypertension, cystocele	CT	Laparotomy	N/A	N/A	Colon	No	N/A	Death
7	Shekar et al. [34]	78/F	Right flank pain	N/A	CT	Laparoscopy	N/A	Suture (NA), mesh (composite)	Renal pelvis, ureter	No	None	No recurrence at 1 month
8	Daha et al. [35]	24/F	Chest pain, abdominal pain	Unremarkable	CT	Laparoscopy	10 × 10	Mesh (N/A)	Small bowel, colon, mesentery, kidney	No	None	N/A
9	Toda et al. [36]	72/M	Nausea	Adipositas	CT	Thoracotomy	8 × 5	Suture (N/A)	Intra-abdominal fat	No	None	No recurrence at 4 months
10	Hunter et al. [29]	69/F	Right-sided chest pain, abdominal pain	N/A	CT	Robotic-assisted thoracoscopy	N/A	Suture (NA)	Intra-abdominal fat	No	None	No recurrence at 4 months
11	Hunter et al. [29]	48/F	Abdominal pain	N/A	CT	Robotic-assisted thoracoscopy	N/A	Suture (NA)	Liver	No	None	No recurrence at 12 months
12	Moro et al. [7]	89/F	Dyspnea, abdominal pain	Right femoral hernia, uterine myoma	CT	Laparotomy	3	Suture (A)	Small bowel	Small bowel	None	N/A
13	Ayane et al. [47]	35/F	Right-sided chest pain, abdominal pain	Unremarkable	CT	Laparotomy	N/A	N/A	Small bowel, colon	Colon	None	No recurrence at 9 months
14	Jambhekar et al. [28]	74/M	Nausea, diarrhea, decreased appetite	Benign prostatic hypertrophy	CT	Robotic-assisted laparoscopy	6 × 8	Suture (NA)	Colon	Colon	None	No recurrence at 21 months
15	Kohli et al [33]	22/M	Right-sided chest pain, dyspnea, right shoulder pain	Asthma	CT	Laparoscopy, thoracotomy	10	Mesh (composite)	Small bowel, colon	No	Wound infection (PO)	No recurrence at 0.5 months
16	Ohtsuka and Suzuki [41]	89/F	Dyspnea, abdominal pain, nausea	Hypertension	CT	Laparotomy	0.45 × 0.3	Suture (NA)	Small bowel, colon	No	Pneumonia (PO)	No recurrence at 24 months
17	Kikuchi et al. [48]	76/F	None	Hypertension, uterine cervical cancer, cholangiectasis	CT	Laparoscopy	N/A	None	Liver	N/A	None	No recurrence at 3 months
18	Watanabe et al. [38]	65/F	Dyspnea, abdominal pain	Rheumatoid arthritis	CT	Laparotomy	5	Suture (A)	Liver, colon	Colon	Right thoracic cavity abscess (HR)	No recurrence at 56 months
19	Dos Santos-Netto et al. [42]	45/F	Abdominal distension, jaundice with itching, choluria	Spontaneous abortion	CT	Thoraco-phreno-laparotomy	10	Mesh (NA)	Liver, kidney, colon	No	Pneumonia, sepsis (PO)	Death
20	Chen et al. [27]	80/F	Dyspnea, abdominal pain, nausea, decreased appetite	Hypertension, ischemic heart disease, peptic ulcer disease	CT	Robotic-assisted laparoscopy	3.6	Mesh (composite)	Kidney	No	Hydronephrosis (HR)	No recurrence at 6 months
21	Hatzidakis et al. [24]	86/F	Right flank pain	N/A	CT	Percutaneous nephrostomy, external-internal nephroureteral double pigtail	N/A	None	N/A	N/A	Hydronephrosis, pleural effusion (HR)	N/A
22	Onuk et al. [25]	72/F	Chest pain, dyspnea, difficulty in urination, back pain, fatigue	Unremarkable	CT	None	N/A	None	Bowel loops, kidney	No	Ureteropelvic junction obstruction (HR)	No recurrence at 6 months
23	Choe and Kahler [49]	61/M	Right-sided chest pain	COPD	CT	Laparotomy	N/A	Mesh (NA)	Colon	N/A	None	No recurrence at 12 months
24	Frisoni et al. [50]	39/M	Dyspnea, abdominal pain, shoulder pain	N/A	CT, MRI	Thoracoscopy	N/A	Suture (NA), mesh (NA)	Colon	No	None	No recurrence at 6 months
25	Wenzel-Smith [16]	40/F	Dyspnea, abdominal pain, nausea	Asthma, hypertension	X-ray	Laparotomy	N/A	Suture (A)	Small bowel	Small bowel	Fecothorax, necrotic small bowel (HR)	No recurrence at 1 month
26	Costa Almeida et al. [51]	49/F	Dyspnea	Unremarkable	CT	Laparotomy	6 × 3	Suture (NA)	Small bowel, colon	No	None	No recurrence at 24 months
27	Shenoy and Johri [52]	Elderly man/M	Dyspnea, abdominal pain, nausea	N/A	CT	Laparotomy	3 × 2	Suture (NA)	Small bowel	No	None	N/A
28	Patle et al. [40]	50/F	Dyspnea, right-sided thoracic pain, abdominal pain	Hypertension, diabetes	CT	Laparoscopy	10 × 8	Suture (NA), mesh (NA)	Colon, kidney	No	Pleural effusion (HR)	N/A
29	Baek et al. [20]	53/M	Abdominal pain	Unremarkable	CT	Laparotomy	12 × 10	Mesh (NA)	Liver, gallbladder, colon, omentum	No	Duodenal ulcer perforation	No recurrence at 12 months
30	Deb [21]	54/M	Abdominal pain, nausea	N/A	CT	Laparotomy, thoracotomy	N/A	Mesh (NA)	Stomach, liver, gallbladder, small bowel, omentum	No	Acute cholecystitis	No recurrence at 12 months
31	Kumar et al. [26]	37/F	Fecoptysis	N/A	CT	N/A	N/A	N/A	Colon	No	Broncho-pleuro-colonic fistula (HR)	N/A
32	Agrafiotis et al. [30]	52/F	Abdominal pain, nausea	Unremarkable	CT	Laparoscopy	N/A	Clips, mesh (NA)	Small bowel, colon	No	None	No recurrence at 9 months
33	Sofi et al. [53]	23/F	Dyspnea	Unremarkable	CT	Thoracotomy	10 × 7	N/A	Small bowel, colon	No	None	N/A
34	Granier et al. [43]	54/F	Dyspnea	Rheumatoid arthritis	CT	Laparotomy	N/A	N/A	Small bowel, colon	Colon, small bowel	Perforation of caecum, septic shock (HR)	Death
35	Trivedi et al. [22]	71/M	Dyspnea	Asthma, hypertension, benign prostatic hypertrophy	CT	None	N/A	None	Small bowel, stomach, colon, pancreas	No	None	No recurrence at 12 months
36	Laaksonen et al. [31]	38/F	Abdominal pain	N/A	CT	Laparoscopy, thoracotomy	10	Suture (N/A), mesh (A)	Liver, colon, omentum	No	None	N/A
37	Fraser et al. [54]	75/F	Cough	N/A	CT	Laparoscopy, thoracoscopy	8 × 5	Mesh (NA)	Small bowel, colon, kidney	No	None	No recurrence at 11 months
38	Terzi et al. [18]	70/F	Dyspnea	N/A	MRI	Laparoscopy, thoracoscopy	4	Mesh (NA)	Colon, omentum	N/A	None	N/A
39	Kavanagh et al. [44]	76/M	Dyspnea, abdominal distension, nausea	Laryngeal carcinoma	CT	Laparotomy	N/A	Suture (NA)	Liver, colon	Colon	Postoperative cerebrovascular accidents (PO)	Death
40	Katsenos et al. [19]	45/M	Right-sided chest pain	Smoking	MRI	None	N/A	None	Liver	No	None	N/A
41	Goh et al. [55]	27/M	Dyspnea	N/A	CT	Thoracotomy	N/A	Mesh (NA)	Liver	No	None	N/A
42	Luo et al. [56]	50/F	Chest pain, cough	N/A	CT	Thoracotomy	N/A	N/A	Liver	N/A	None	N/A
43	Owen et al. [17]	35/F	Abdominal pain, nausea	Unremarkable	Clinically	Laparotomy	N/A	Suture (A)	Small bowel	Small bowel	None	N/A
44	Rosen et al. [57]	50/M	Dyspnea	Unremarkable	CT	Laparoscopy	2 × 2	Mesh (NA)	Intra-abdominal fat	N/A	None	No recurrence at 6 months

*M* male, *F* female, *COPD* chronic obstructive pulmonary disease, *CT* computed tomography, *MRI* magnetic resonance imaging, *N/A* not available, *A* absorbable, *NA* non-absorbable, *HR* hernia-related, *PO* postoperative

### Data extraction

The following variables were extracted from each article: patient’s age, sex, chief complaint, and past medical history, diagnostic imaging technique, surgical approach and procedure, size of hernia orifice, type of defect closure, herniated organ, need for bowel resection, hernia-related or postoperative complications, and patient’s outcome.

### Methodological quality assessment

A modified version of the Newcastle–Ottawa Scale (NOS) designed for case reports was applied to assess the methodological quality of all included studies [15]. The tool consists of eight questions, categorized in four domains: selection, ascertainment, causality, and reporting. Three questions were excluded as they were irrelevant to the included studies. Questions were answered by a binary response (yes or no) independently by JPR and SR. Finally, an overall judgement about the study quality was performed. Studies were of good quality (low risk of bias) when all five questions were fulfilled, moderate when four were fulfilled, and low (high risk of bias) when three or fewer were fulfilled. No disagreements were found between the reviewers.

### Statistical analysis

Statistical analysis was performed using R software, GraphPad Prism (version 9.00 for Windows, GraphPad Software, La Jolla, CA, USA, www.graphpad.com), and Microsoft Office Excel 365. Normality was measured using the Shapiro–Wilk normality test and data were reported accordingly.

## Results

**Fig. 1:**
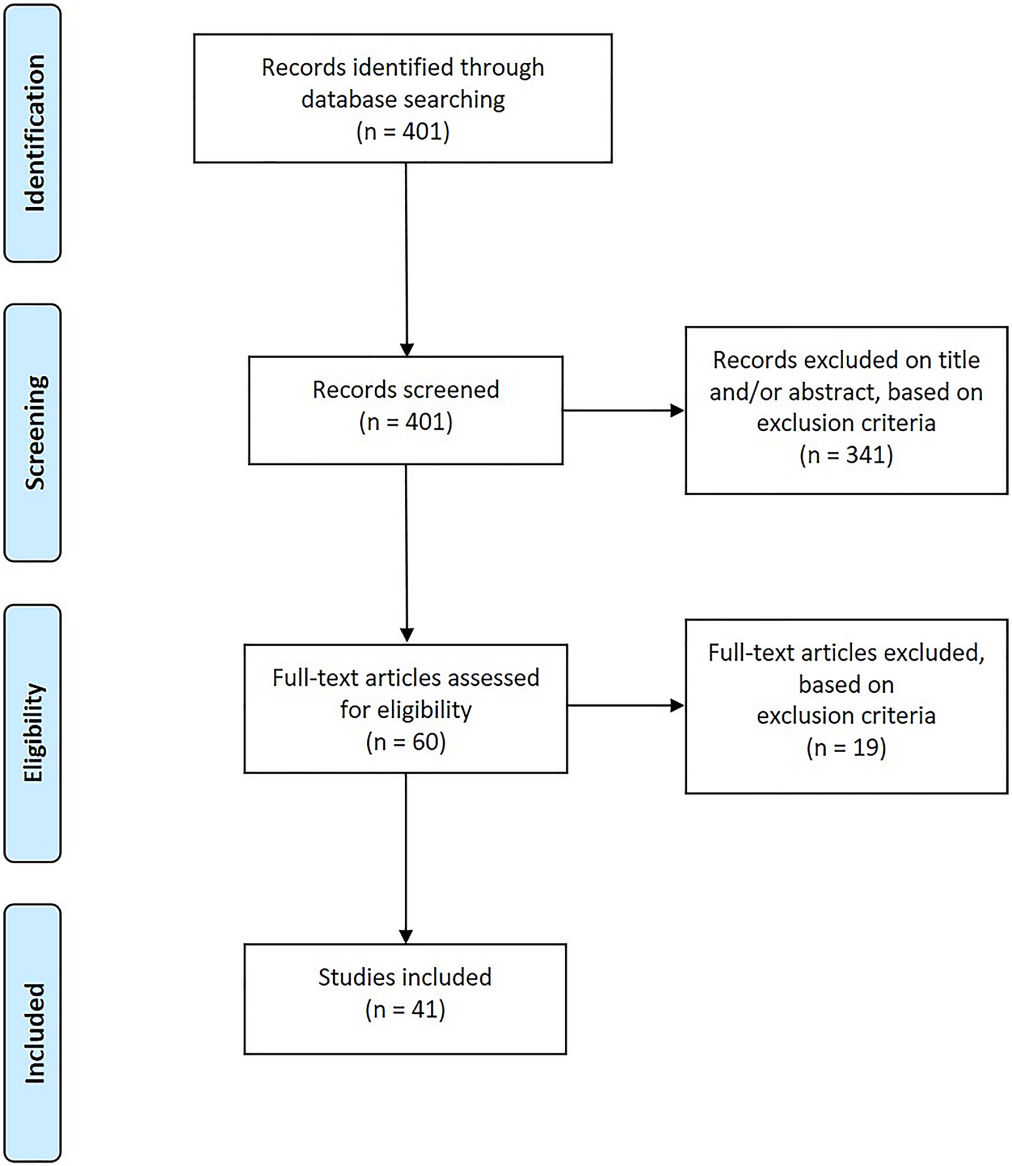
PRISMA flowchart of study selection

Two databases were searched resulting in 401 records. In total, 341 studies were excluded on title and/or abstract and 19 studies after full-text screening because of missing inclusion or defined exclusion criteria (trauma *n* = 2, patient’s age < 18 years *n* = 4, other type of hernia *n* = 2, pregnancy-associated hernias *n* = 1, missing full text *n* = 1, non-English literature *n* = 1, and others *n* = 8). After detailed screening only 41 studies of adult right-sided Bochdalek hernias were found to be eligible for this systematic review. The study selection process is shown in the PRISMA flowchart (Fig. 1). No overlapping study populations were identified. The characteristics of the included studies are presented in Table 1. The results of methodological quality assessment are shown in Supplementary Table 1. Overall, eight studies (18%) were judged as good, 14 (32%) as moderate, and 22 (50%) as low quality.

### Patients’ baseline characteristics

**Fig. 2:**
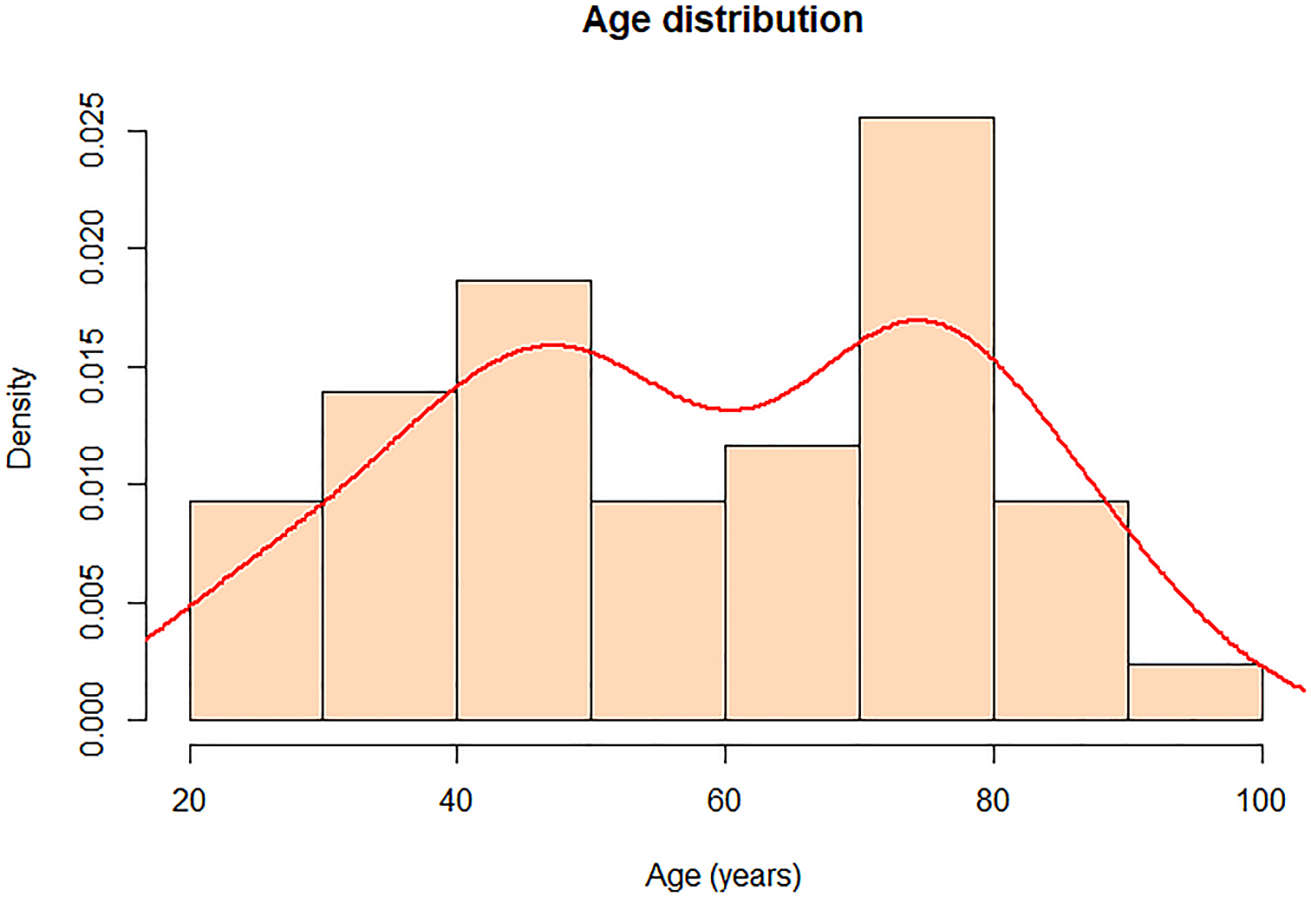
Age distribution in patients with right-sided Bochdalek hernias; *x*-axis shows age in years, *y*-axis shows density. Density curve is shown in red. Two peak ages 40 to 50 and 70 to 80 years can be detected

Mean age of the included patients was 58 years ranging from 22 to 92 years. Peak ages for right-sided Bochdalek hernias were 40 to 50 and 70 to 80 years (Fig. 2). 61% of the patients were women (*n* = 27), 39% (*n* = 17) were men. Almost half of the patients (*n* = 21) presented with a chief complaint of dyspnea. Abdominal pain was the second most common symptom (*n* = 19, 43%) whereas chest pain and nausea were only reported in 10 cases (23%). A minority of patients suffered from shoulder and/or flank pain (*n* = 8, 18%). Five patients (11%) suffered from a pulmonary disease like chronic obstructive pulmonary disease (COPD) or asthma. Furthermore, analysis of the past medical history revealed hypertension (*n* = 7, 16%) and diabetes (*n* = 2, 5%) in some further patients. Ten cases (23%) had an unremarkable history (Table 1).

### Diagnostic imaging

Final diagnosis of right-sided Bochdalek hernias in adulthood was mostly performed using computed tomography (CT) scan (*n* = 39, 89%). In contrast, only two patients (4%) were diagnosed clinically or via X-ray [16, 17]. In two cases (5%) magnetic resonance imaging (MRI) led to the final diagnosis [18, 19].

### Herniated organ and size of hernia orifice

In most cases, herniation of the following organs was found: colon (*n* = 23, 52%), small bowel (*n* = 19, 43%), liver (*n* = 12, 27%), and kidney (*n* = 8, 18%). Single cases described herniation of other intraabdominal organs like gallbladder [20, 21] or pancreas [22]. Only half of the included studies described the size of the hernia orifice (*n* = 23, 52%). 65% (*n* = 15) of them calculated a minimum of a two-dimensional orifice varying from 0.54 × 0.3 cm to 12 × 10 cm.

### Treatment strategy and surgical approach

Almost all reported cases (*n* = 38, 86%) were treated surgically. One patient was treated with a right-sided double-J ureteral stent due to preoperative hydronephrosis and outpatient surgical repair of the hernia was recommended. Further information about the follow-up was missing [23]. In one case, percutaneous nephrostomy was performed as the patient was not fit enough to undergo surgery. Further details were not described. She was finally discharged with an external-internal nephroureteral double pigtail for treatment of hernia-related hydronephrosis [24]. Three patients underwent non-interventional management of their Bochdalek hernias. One of them was treated conservatively. Surgical repair was denied due to fear of the operative risks. He did not report any symptoms after 12 months [22]. In one further case, the patient refused any surgical repair and denied any problems 6 months later [25]. In the third case, symptoms resolved a few days after initial presentation in the hospital. A follow-up was not described [19]. No treatment strategy of the diaphragmatic defect was reported in one further case [26]. Most patients underwent an abdominal approach for surgical repair. Laparotomy was performed in 34% (*n* = 15) and a laparoscopy in 34% (*n* = 15) of all cases. Two patients (5%) underwent robotic-assisted laparoscopy [27, 28]. Only in 16% (*n* = 7) a thoracotomy, and in 14% (*n* = 6) a thoracoscopy was performed. Two patients (5%) were operated by robotic-assisted thoracoscopy [29]. Seven patients (16%) underwent a thoracoabdominal approach. If a bowel resection was required, an open approach was more common (nine of ten cases). In one case colon resection was performed by robotic-assisted laparoscopy [28]. No data about operative time and surgical experience were available.

### Type of defect closure

The most common type of repair was done by direct diaphragmatic sutures in 20 cases (45%) followed by mesh-augmented defect-closure in 19 cases (43%). A combination of mesh and suture for diaphragmatic defect repair was used in 16% (*n* = 7). One case reported a combination of clips and mesh [30]. Six cases (14%) did not report the type of closure. Most patients with a mesh received a non-absorbable mesh (*n* = 13, 68%). In two cases diaphragmatic defect was repaired by an absorbable mesh (11%) [31, 32]. A composite mesh was used in three patients (16%) [27, 33, 34]. One case did not report the type of mesh [35]. The suture type was mostly non-absorbable (*n* = 13, 65%) followed by absorbable sutures (*n* = 4, 20%). Three cases (15%) did not describe the type of suture [31, 32, 36].

### Complication

Twenty-five patients (57%) did not show any hernia or intervention-related complications. One case did not report any information about difficulties [37]. Complications mostly included a hydronephrosis (*n* = 4, 9%) due to herniation of ureter or kidney [6, 7, 18, 19]. In two of these patients hydronephrosis resolved after surgical treatment [18, 19], whereas two other cases did not report any follow-up [6, 7]. In eight patients (18%) right-sided Bochdalek hernias were associated with thoracic complications: abscess formation [38], lung empyema [39], broncho-pleuro-colonic fistula [26], pleural effusion [24, 40], fecothorax [16], and pneumonia [41, 42]. One patient underwent a surgical approach to the chest and abdomen [42]. Laparotomy was performed in four cases [16, 38, 39, 41], laparoscopy in one case [30]. One patient underwent urological treatment [24] and one further case did not report the surgical procedure [26]. Other hernia-related complications included bowel ischemia (*n* = 2, 4%) [16, 32] and bowel perforation (*n* = 2, 4%) [20, 43]. Postoperative complications included wound infection (*n* = 1, 2%) [33] and sepsis (*n* = 1, 2%) [42]. In ten cases (23%) colon and/or small bowel resection was performed due to mentioned bowel complications.

### Outcome

Four patients (9%) died after the surgical procedure due to pneumonia and sepsis [42], sequelae of bowel perforation [43], or cerebrovascular incidents [44]. One case did not report any specific reason for postoperative death [37]. Median follow-up was 9 months with no recurrence, but more than one-third of the published cases (*n* = 16) did not report any kind of follow-up at all.

### Diagnostic, therapeutic, and follow-up management algorithm

**Fig. 3:**
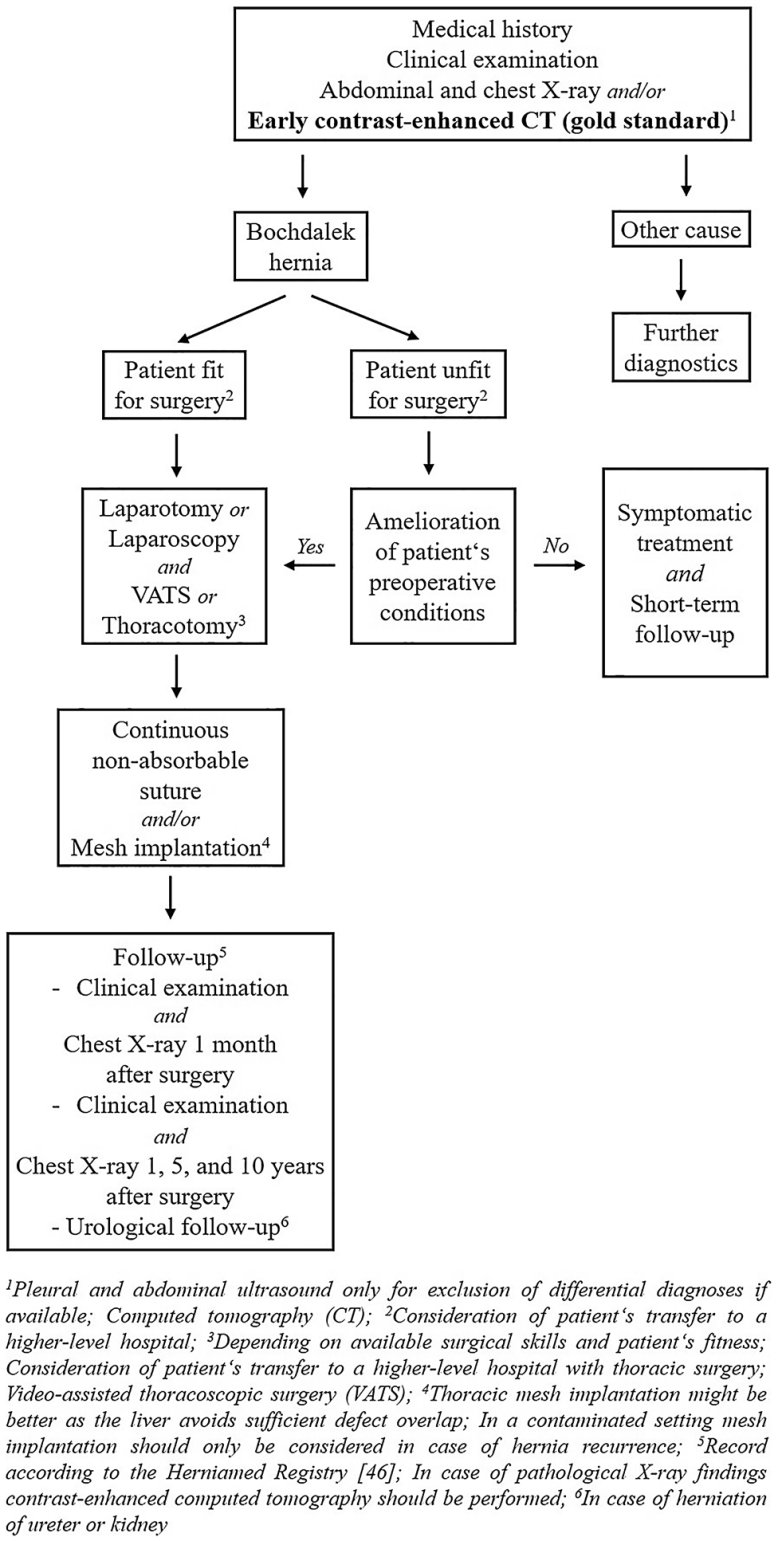
Diagnostic, therapeutic, and follow-up management pathway of adult Bochdalek hernias

A clear clinical guideline and/or algorithm for right-sided Bochdalek hernias in adults is missing so far. Therefore, based upon our comprehensive literature review, we present the current diagnostic, therapeutic, and follow-up management pathway (Fig. 3).

## Discussion

This systematic review demonstrates that there are only very limited studies available investigating the diagnostics, treatment, and follow-up of right-sided Bochdalek hernias in adulthood. However, this review has several strengths. The PRISMA guideline [12] was followed, a snowball search was conducted, and screening and quality assessment of records was performed by two independent reviewers. Most publications which were identified are of moderate to low methodological quality as all of them were case reports. Most reports did not report defect size, surgical treatment details, or long-term follow-up data.

The following conclusions can be conducted from this systematic review. Right-sided Bochdalek hernia in adulthood is a rare surgical entity overall and in numerous cases an emergency. Patients mostly present with dyspnea and/or abdominal pain. Its masquerading clinical appearance requires rapid diagnostic imaging (gold standard CT) following surgical repair of each symptomatic and/or complicated hernia to decrease high mortality and morbidity rates (Fig. 3). Pregnancy or a medical history of pulmonary diseases may increase intraabdominal pressure and, therefore, precipitate the development of right-sided Bochdalek hernias in adults. Due to missing long-term follow-up data and its rarity, the adequate surgical procedure is unclear so far and only based on a case-by-case basis.

Any clinical or imaging signs of acute incarceration, perforation and/or ileus must lead to emergent surgical management. If surgery is significantly delayed in these situations or if patients are not fit enough to undergo emergent surgery, symptomatic therapy with a highly complicated outcome remains the online available option.

A laparotomy enables sufficient management of visceral complications like perforation, evaluation of bowel perfusion, and management of potential abdominal contamination. Contrarily, only limited exposure of the chest cavity risks not to detect a concomitant pneumothorax, pleural effusion or empyema, or other thoracic-related complications [45]. Repair of the hernia orifice can be easier performed by thoracotomy, particularly in right-sided Bochdalek hernias, in which the liver may mask the diaphragmatic defect [7]. A thoracoabdominal approach permits evaluation and adequate surgical treatment of both cavities and should only be performed if necessary. If so, open approach should be combined with a minimally invasive one depending on technical feasibility. Minimally invasive surgery may be associated with technical difficulties. Necessary expertise in advanced thoracoscopy is often not available in general surgical departments, in particular in the often nocturnal emergency setting. In addition, patients with delayed presentation may not be fit enough for thoracoscopy and/or laparoscopy regarding the risk of anesthesia. Patient’s transfer to a higher-level hospital must always be considered if expertise in complex surgery, including two-cavity and thoracic surgery or intensive care is not available. In patients who undergo abdominal approach for right-sided Bochdalek repair it remains unclear if a simultaneous thoracic approach via video-assisted thoracoscopic surgery (VATS) or thoracotomy following extensive intrathoracic evaluation and lavages would prevent later development of thoracic complications. Diaphragmatic defects were mostly repaired by direct diaphragmatic sutures and mesh-augmentation. For better defect augmentation we strongly recommend non-absorbable suture as well as non-absorbable mesh in accordance with the general principles of hernia surgery. In case of intraabdominal or intrathoracic contamination mesh implantation should only be considered in case of hernia recurrence. The use of the hernia sac to cover the mesh against the respective organs and anti-adhesive meshes could reduce postoperative adhesions.

This review has several limitations. First, most publications, which were identified are of moderate or low methodological quality as all of them were case reports with a low level of evidence. Due to their retrospective character, there might be a selection/publication bias. Second, most reports did not report all relevant details like defect size or surgical treatment. Besides, follow-up was often insufficient or mainly short-term complicating further diagnostic and treatment strategy development. Therefore, there is urgent need for standardized reporting of right-sided Bochdalek hernias in adulthood [46]. Finally, the small number of patients yields the risk to over-interpret the data.

Based upon our literature review, we hereby present the current follow-up algorithm with regular follow-up visits including imaging up to 10 years after surgery. In cases of herniation of ureter and/or kidney, urological consultation is mandatory (Fig. 3). Hernia recurrence after surgical repair has not been described so far, but recurrence obviously must be expected in at least some cases. Current numbers of recurrence should be viewed with caution due to insufficient follow-up data.

## Conclusion

This systematic review underlined that studies investigating diagnostic algorithm, treatment strategy, and follow-up of right-sided Bochdalek hernias in adulthood are of low methodological quality to date. Despite their overall low incidence, this type of hernia tends to occur more frequently in middle-aged and older women mostly presenting with abdominal pain and dyspnea. A rapid and accurate diagnosis following surgical repair is mandatory. Based on the literature available a comprehensive suggested pathway for the emergency management of patients presenting with acutely complicated Bochdalek hernia was developed. Due to the rarity of these hernias, high-quality studies with a sufficient investigation as an own entity are almost impossible to conduct. Instead, observational studies could provide more evidence-based insights. Therefore, data about this entity, patients’ characteristics, and their medical work-up must be reported in a standardized manner and/or common database.

## Supplementary Information

Below is the link to the electronic supplementary material.

Supplementary file 1 (DOCX 48 KB)

## References

[1] Keijzer R, Puri P (2010). Congenital diaphragmatic hernia. Semin Pediatr Surg.

[2] Kotecha S, Barbato A, Bush A, Claus F, Davenport M, Delacourt C, Deprest J, Eber E, Frenckner B, Greenough A, Nicholson AG, Antón-Pacheco JL, Midulla F (2012). Congenital diaphragmatic hernia. Eur Respir J.

[3] Leeuwen L, Fitzgerald DA (2014). Congenital diaphragmatic hernia. J Paediatr Child Health.

[4] Sperling JD, Sparks TN, Berger VK, Farrell JA, Gosnell K, Keller RL, Norton ME, Gonzalez JM (2018). Prenatal diagnosis of congenital diaphragmatic hernia: does laterality predict perinatal outcomes?. Am J Perinatol.

[5] Kardon G, Ackerman KG, McCulley DJ, Shen Y, Wynn J, Shang L, Bogenschutz E, Sun X, Chung WK (2017). Congenital diaphragmatic hernias: from genes to mechanisms to therapies. Dis Model Mech.

[6] Mullins ME, Stein J, Saini SS, Mueller PR (2001). Prevalence of incidental Bochdalek’s hernia in a large adult population. AJR Am J Roentgenol.

[7] Moro K, Kawahara M, Muneoka Y, Sato Y, Kitami C, Makino S, Nishimura A, Kawachi Y, Gabriel E, Nikkuni K (2017). Right-sided Bochdalek hernia in an elderly adult: a case report with a review of surgical management. Surg Case Rep.

[8] Brown SR, Horton JD, Trivette E, Hofmann LJ, Johnson JM (2011). Bochdalek hernia in the adult: demographics, presentation, and surgical management. Hernia.

[9] Mah VK, Zamakhshary M, Mah DY, Cameron B, Bass J, Bohn D, Scott L, Himidan S, Walker M, Kim PC (2009). Absolute vs relative improvements in congenital diaphragmatic hernia survival: what happened to “hidden mortality”. J Pediatr Surg.

[10] Colvin J, Bower C, Dickinson JE, Sokol J (2005). Outcomes of congenital diaphragmatic hernia: a population-based study in western Australia. Pediatrics.

[11] Puligandla PS, Skarsgard ED, Offringa M, Adatia I, Baird R, Bailey M, Brindle M, Chiu P, Cogswell A, Dakshinamurti S, Flageole H, Keijzer R, McMillan D, Oluyomi-Obi T, Pennaforte T, Perreault T, Piedboeuf B, Riley SP, Ryan G, Synnes A, Traynor M (2018). Diagnosis and management of congenital diaphragmatic hernia: a clinical practice guideline. CMAJ.

[12] Moher D, Liberati A, Tetzlaff J, Altman DG (2009). Preferred reporting items for systematic reviews and meta-analyses: the PRISMA statement. PLoS Med.

[13] Rout S, Foo FJ, Hayden JD, Guthrie A, Smith AM (2007). Right-sided Bochdalek hernia obstructing in an adult: case report and review of the literature. Hernia.

[14] Greenhalgh T, Peacock R (2005). Effectiveness and efficiency of search methods in systematic reviews of complex evidence: audit of primary sources. BMJ.

[15] Murad MH, Sultan S, Haffar S, Bazerbachi F (2018). Methodological quality and synthesis of case series and case reports. BMJ Evid Based Med.

[16] Wenzel-Smith G (2013). Posterolateral diaphragmatic hernia with small-bowel incarceration in an adult. S Afr J Surg.

[17] Owen ME, Rowley GC, Tighe MJ, Wake PN (2007). Delayed diagnosis of infarcted small bowel due to right-sided Bochdalek hernia. Ann R Coll Surg Engl.

[18] Terzi A, Tedeschi U, Lonardoni A, Furia S, Benato C, Calabrò F (2008). A rare cause of dyspnea in adult: a right Bochdalek’s hernia-containing colon. Asian Cardiovasc Thorac Ann.

[19] Katsenos S, Kokkonouzis I, Lachanis S, Psathakis K (2008). Right-sided Bochdalek hernia presenting as a solitary pulmonary nodule. Radiol Case Rep.

[20] Baek SJ, Kim J, Lee SH (2012). Hepatothorax due to a right diaphragmatic rupture related to duodenal ulcer perforation. World J Gastroenterol.

[21] Deb SJ (2011). Massive right-sided Bochdalek hernia with two unusual findings: a case report. J Med Case Rep.

[22] Trivedi PJ, Canavan J, Holloway C, Slater A, Travis S (2010). An unusual case of dyspnoea in an elderly man. BMJ Case Rep.

[23] Nassiri N, Maas M, Asanad K, Hwang D, Duddalwar V, Bhanvadia S (2020). An 82-year-old female with chest pain radiating to the back and flank. Urol Case Rep.

[24] Hatzidakis A, Kozana A, Glaritis D, Mamoulakis C (2014). Right-sided Bochdalek hernia causing septic ureteric obstruction. Percutaneous treatment with placement of a nephroureteral double pigtail. BMJ Case Rep.

[25] Onuk Ö, Taş T, Şentürk AB, Sinanoğlu O, Balcı MB, Çelik O, Nuhoğlu B (2014). Right-sided Bochdalek hernia with intrathoracic ectopic kidney in an advanced-age adult: a case report. Urol Int.

[26] Kumar M, Chandra A, Kumar S (2011). Right-sided diaphragmatic hernia complicated with broncho-pleuro-colonic fistula presenting as fecoptysis. BMJ Case Rep.

[27] Chen B, Finnerty BM, Schamberg NJ, Watkins AC, DelPizzo J, Zarnegar R (2015). Transabdominal robotic repair of a congenital right diaphragmatic hernia containing an intrathoracic kidney: a case report. J Robot Surg.

[28] Jambhekar A, Robinson S, Housman B, Nguyen J, Gu K, Nakhamiyayev V (2018). Robotic repair of a right-sided Bochdalek hernia: a case report and literature review. J Robot Surg.

[29] Hunter LM, Mozer AB, Anciano CJ, Oliver AL, Iannettoni MD, Speicher JE (2019). Robotic-assisted thoracoscopic repair of right-sided Bochdalek hernia in adults: a two-case series. Innovations.

[30] Agrafiotis AC, Kotzampassakis N, Boudaka W (2011). Complicated right-sided Bochdalek hernia in an adult. Acta Chir Belg.

[31] Laaksonen E, Silvasti S, Hakala T (2009). Right-sided Bochdalek hernia in an adult: a case report. J Med Case Rep.

[32] Lau NS, Crawford M, Sandroussi C (2020). Surgical management of symptomatic right-sided Bochdalek hernias in adults: when is a minimally invasive approach appropriate?. ANZ J Surg.

[33] Kohli N, Mitreski G, Yap CH, Leong M (2016). Massive symptomatic right-sided Bochdalek hernia in an adult man. BMJ Case Rep.

[34] Shekar PA, Reddy D, Kochhar G, Dumra A, Ks S (2020). Herniation of the right renal pelvis through a posterolateral diaphragmatic defect (Bochdalek hernia). Urology.

[35] Daha SK, Karn A, Shrestha N, Shrestha N, Paudyal S, Giri N (2019). A female with right-sided thoracic kidney with Bochdalek hernia: a case report. JNMA J Nepal Med Assoc.

[36] Toda M, Yamamoto A, Iwata T (2019). Right-sided Bochdalek hernia containing retroperitoneal fat in the elderly: report of a case. Surg Case Rep.

[37] Rocha Paiva D, Casanova D, Martins H, Cerqueira M, Formigo M, Miranda O, Cotter J (2020). A rare cause of dyspnoea: right-sided Bochdalek hernia in an adult. Eur J Case Rep Intern Med.

[38] Watanabe M, Ishibashi O, Watanabe M, Kondo T, Ohkohchi N (2015). Complicated adult right-sided Bochdalek hernia with Chilaiditi’s syndrome: a case report. Surg Case Rep.

[39] Gupta S, Warrell D, Smith L, Williams GL (2020). Strangulated right-sided diaphragmatic hernia presenting and treated as lung empyema: beware of the differential diagnosis. BMJ Case Rep.

[40] Patle NM, Tantia O, Prasad P, Das PC, Khanna S (2013). Laparoscopic repair of right sided Bochdalek hernia—a case report. Indian J Surg.

[41] Ohtsuka Y, Suzuki TH (2017). Right-sided Bochdalek hernia in an elderly patient: a case review of adult Bochdalek hernias from 1982 to 2015 in Japan. Acute Med Surg.

[42] dos Santos-Netto JM, Oliveira CV, Sousa MG (2015). Right-sided Bochdalek hernia in adult associated with cholestatic syndrome: case report. Arq Bras Cir Dig.

[43] Granier V, Coche E, Hantson P, Thoma M (2010). Intrathoracic caecal perforation presenting as dyspnea. Case Rep Med.

[44] Kavanagh DO, Ryan RS, Waldron R (2008). Acute dyspnoea due to an incarcerated right-sided Bochdalek’s hernia. Acta Chir Belg.

[45] Suzuki T, Okamoto T, Hanyu K, Suwa K, Ashizuka S, Yanaga K (2014). Repair of Bochdalek hernia in an adult complicated by abdominal compartment syndrome, gastropleural fistula and pleural empyema: report of a case. Int J Surg Case Rep.

[46] Stechemesser B, Jacob DA, Schug-Paß C, Köckerling F (2012). Herniamed: an internet-based registry for outcome research in hernia surgery. Hernia.

[47] Ayane GN, Walsh M, Shifa J, Khutsafalo K (2017). Right congenital diaphragmatic hernia associated with abnormality of the liver in adult. Pan Afr Med J.

[48] Kikuchi S, Nishizaki M, Kuroda S, Kagawa S, Fujiwara T (2016). A case of right-sided Bochdalek hernia incidentally diagnosed in a gastric cancer patient. BMC Surg.

[49] Choe CH, Kahler JJ (2014). Herniation of the lung: a case report. J Emerg Med.

[50] Frisoni R, Germain A, Ayav A, Brunaud L, Bresler L (2014). Thoracoscopic treatment of a right Bochdalek hernia in an adult (with video). J Visc Surg.

[51] Costa Almeida CE, Reis LS, Almeida CM (2013). Adult right-sided Bochdalek hernia with ileo-cecal appendix: Almeida-Reis hernia. Int J Surg Case Rep.

[52] Shenoy KR, Johri G (2013). Congenital right Bochdalek hernia presenting as emergency in old age: a case report. Indian J Surg.

[53] Sofi FA, Ahmed SH, Dar MA, Nabhi DG, Mufti S, Bhat MA, Tabassum PN (2011). Nontraumatic massive right-sided Bochdalek hernia in an adult: an unusual presentation. Am J Emerg Med.

[54] Fraser JD, Craft RO, Harold KL, Jaroszewski DE (2009). Minimally invasive repair of a congenital right-sided diaphragmatic hernia in an adult. Surg Laparosc Endosc Percutan Tech.

[55] Goh BK, Teo MC, Chng SP, Soo KC (2007). Right-sided Bochdalek’s hernia in an adult. Am J Surg.

[56] Luo HF, Lei T, Wang HJ, Tan G, Wang ZY (2007). Non-traumatic diaphragmatic hernia of the liver in an adult: a case report. Hepatobiliary Pancreat Dis Int.

[57] Rosen MJ, Ponsky L, Schilz R (2007). Laparoscopic retroperitoneal repair of a right-sided Bochdalek hernia. Hernia.

