# Supplementary material for: A systematic review on diagnostics and surgical treatment of adult right-sided Bochdalek hernias and presentation of the current management pathway
Source: Hernia. 2021 Jul 3;26(1):47–59. doi: 10.1007/s10029-021-02445-1 (PMC8881253; doi:10.1007/s10029-021-02445-1)
Supplement: Supplementary file 1 — (DOCX 48 KB) [file 10029_2021_2445_MOESM1_ESM.docx]

**Supplementary Tab. 1 Methodological quality assessment.** Question 1: Question 1 was considered as “yes” if no information about further cases was described. Question 2: Adequately described exposure/diagnosis was considered if computed tomography scan or or magnetic resonance imaging was performed. Question 3: Correctly described postoperative outcome was considered if data about postoperative complications was reported (“yes” or “no”, if “yes” type of complication). Question 4: Correctly ascertained outcome was considered if minimum follow-up of six months or patient’s death was reported. Question 5: Details were considered as sufficient if all patient’s characteristic (Table 1) were reported. Low=three or fewer questions fulfilled, moderate=four questions fulfilled, good=all five questions fulfilled, Yes (Y), No (N)**.**

|  | | **Selection** | **Ascertainment** | | **Causality** | **Reporting** |  |
| --- | --- | --- | --- | --- | --- | --- | --- |
| **No.** | **Reference** | **1. Does the patient represent the whole case of the investigators/ medical center?** | **2. Was the exposure/ diagnosis adequately ascertained?** | **3. Was the postoperative outcome correctly ascertained?** | **4. Was the outcome correctly ascertained?** | **5. Is the case described with sufficient details to allow other investigators to replicate the research or to allow practitioners make inferences related to their own practice?** | **Overall quality assessment** |
| 1 | Gupta  2020  [39] | Y | Y | Y | N | N | low |
| 2 | Lau  2020  [32] | Y | Y | Y | N | N | low |
| 3 | Lau  2020  [32] | Y | Y | Y | N | N | low |
| 4 | Lau  2020  [32] | Y | Y | Y | Y | Y | good |
| 5 | Nassiri 2020  [23] | Y | Y | Y | N | N | low |
| 6 | Rocha Paiva  2020  [37] | Y | Y | N | Y | N | low |
| 7 | Shekar 2020  [34] | Y | Y | Y | N | N | low |
| 8 | Daha  2019  [35] | Y | Y | Y | N | N | low |
| 9 | Toda  2019  [36] | Y | Y | Y | N | Y | moderate |
| 10 | Hunter 2019  [29] | Y | Y | Y | N | N | low |
| 11 | Hunter 2019  [29] | Y | Y | Y | Y | N | moderate |
| 12 | Moro  2017  [7] | Y | Y | Y | N | N | low |
| 13 | Ayane 2017  [47] | Y | Y | Y | Y | N | moderate |
| 14 | Jambhekar 2017  [28] | Y | Y | Y | Y | Y | good |
| 15 | Kohli  2016  [33] | Y | Y | Y | N | Y | moderate |
| 16 | Ohtsuka 2016  [41] | Y | Y | Y | Y | Y | good |
| 17 | Kikuchi 2016  [48] | Y | Y | Y | N | N | low |
| 18 | Watanabe 2015  [38] | Y | Y | Y | Y | Y | good |
| 19 | Dos Santos-Netto  2015  [42] | Y | Y | Y | Y | Y | good |
| 20 | Chen  2015  [27] | Y | Y | Y | Y | Y | good |
| 21 | Hatzidakis 2014  [24] | Y | Y | Y | N | N | low |
| 22 | Onuk  2014  [25] | Y | Y | Y | Y | N | moderate |
| 23 | Choe  2014  [49] | Y | Y | Y | Y | N | moderate |
| 24 | Frisoni 2014  [50] | Y | Y | Y | Y | N | moderate |
| 25 | Wenzel-Smith  2013  [16] | Y | N | Y | N | N | low |
| 26 | Costa Almeida 2013  [51] | Y | Y | Y | Y | Y | good |
| 27 | Shenoy 2013  [52] | Y | Y | Y | N | N | low |
| 28 | Patle  2013  [40] | Y | Y | Y | N | N | low |
| 29 | Baek  2012  [20] | Y | Y | Y | Y | Y | good |
| 30 | Deb  2011  [21] | Y | Y | Y | Y | N | moderate |
| 31 | Kumar 2011  [26] | Y | Y | Y | N | N | low |
| 32 | Agrafiotis 2011  [30] | Y | Y | Y | Y | N | moderate |
| 33 | Sofi  2011  [53] | Y | Y | Y | N | N | low |
| 34 | Granier 2010  [43] | Y | Y | Y | Y | N | moderate |
| 35 | Trivedi 2010  [22] | Y | Y | Y | Y | N | moderate |
| 36 | Laaksonen 2009  [31] | Y | Y | Y | N | N | low |
| 37 | Fraser  2009  [54] | Y | Y | Y | Y | N | moderate |
| 38 | Terzi  2008  [18] | Y | Y | Y | N | N | low |
| 39 | Kavanagh 2008  [44] | Y | Y | Y | Y | N | moderate |
| 40 | Katsenos 2008  [19] | Y | Y | Y | N | N | low |
| 41 | Goh  2007  [55] | Y | Y | Y | N | N | low |
| 42 | Luo  2007  [56] | Y | Y | Y | N | N | low |
| 43 | Owen  2007  [17] | Y | N | Y | N | N | low |
| 44 | Rosen  2007  [57] | Y | Y | Y | Y | N | moderate |
